# Supplementary material for: Personalized Driver Gene Prediction Using Graph Convolutional Networks with Conditional Random Fields
Source: Biology (Basel). 2024 Mar 14;13(3):184. doi: 10.3390/biology13030184 (PMC10968662; doi:10.3390/biology13030184)
Supplement: Supplementary file 1 [file biology-13-00184-s001.zip › biology-2896050-supplementary.pdf]

Supplementary file

Section S1

We analyzed the identification of rare genes in different samples, including genes that appeared in most samples as well as rare genes that appeared only in individual samples.

On ACC data, ARRB2, CDH2 and APEX1 genes were identified in most samples. ARRB2 has been shown to promote colorectal cancer growth by triggering WTAP [1], its variants are associated with late-onset Alzheimer's disease [2], and is a potential target for PRAD immunotherapy [3]. CDH2 can act with microRNA-124 to exert tumor suppression in non-small cell lung cancer [4], microRNA-194 can target CDH2 inhibits proliferation and metastasis of osteosarcoma cells in vitro and in vivo [5], while CDH2 expression has prognostic significance in glioma [6]. APEX1 is a novel diagnostic and prognostic biomarker for hepatocellular carcinoma [7], and it also serves as a potential biomarker for cholangiocarcinoma [8] and a prognostic marker and potential therapeutic target for osteosarcoma [9]. There are also rare genes that appear only in individual samples, such as PPARGC1A, whose up-regulated expression promotes lung cancer metastasis [10], the HSP90AA1 gene that promotes drug resistance in osteosarcoma through an autophagy-related mechanism [11], the DVL1 gene whose amplification and up-regulation of expression may play a role in the development of human cervical squamous cell carcinoma [12] and human breast cancer [13] through disruption of the Wnt signaling pathway, and the SMARCE1 gene whose loss of function leads to multiple spinal meningiomas [14].

On the KICH data, it basically contains CDK6 as well as ARRB1. CDK family genes are over-expressed in a variety of cancers and are associated with dozens of drug sensitivities [15]. ARRB1 is a potential novel biomarker in kidney cancer [16]. A larger number of genes appear in individual samples, such as FOXO1, a tumor suppressor involved in metastasis and prognosis of kidney cancer [17], and FOXO1 expression is associated with the grading and staging of KICH [18]. RASA1 inhibits the development of KICH by regulating the corresponding microRNA [19]. Up-regulation of ITGB1 expression plays an important role in the development and metastasis of kidney cancer [20]. Mutations in CTNNB1 are although rare, its mutational activation may be involved in the development of KIPAN, which contains KICH, KIRC, and KIRP cancers [21]. MicroRNA-196a regulates the SMAD3 signaling pathway by targeting BRAM1 and promotes KICH cancer cell migration and invasion [22]. PCNA in kidney cancer subtype has some prognostic value [23]. RBX1 protein is associated with cullins and may play a central role in the development of kidney cancer [24]. GNB1 is also associated with kidney disease, and its down-regulation of expression is associated with the progression of KIRC [25].

Section S2

On KICH, we obtained survival analysis map of new biomarker genes. The results are shown in Figure S1. From the results we can see that these novel biomarkers can efficiently distinguish the longer survival group with shorter survival group.

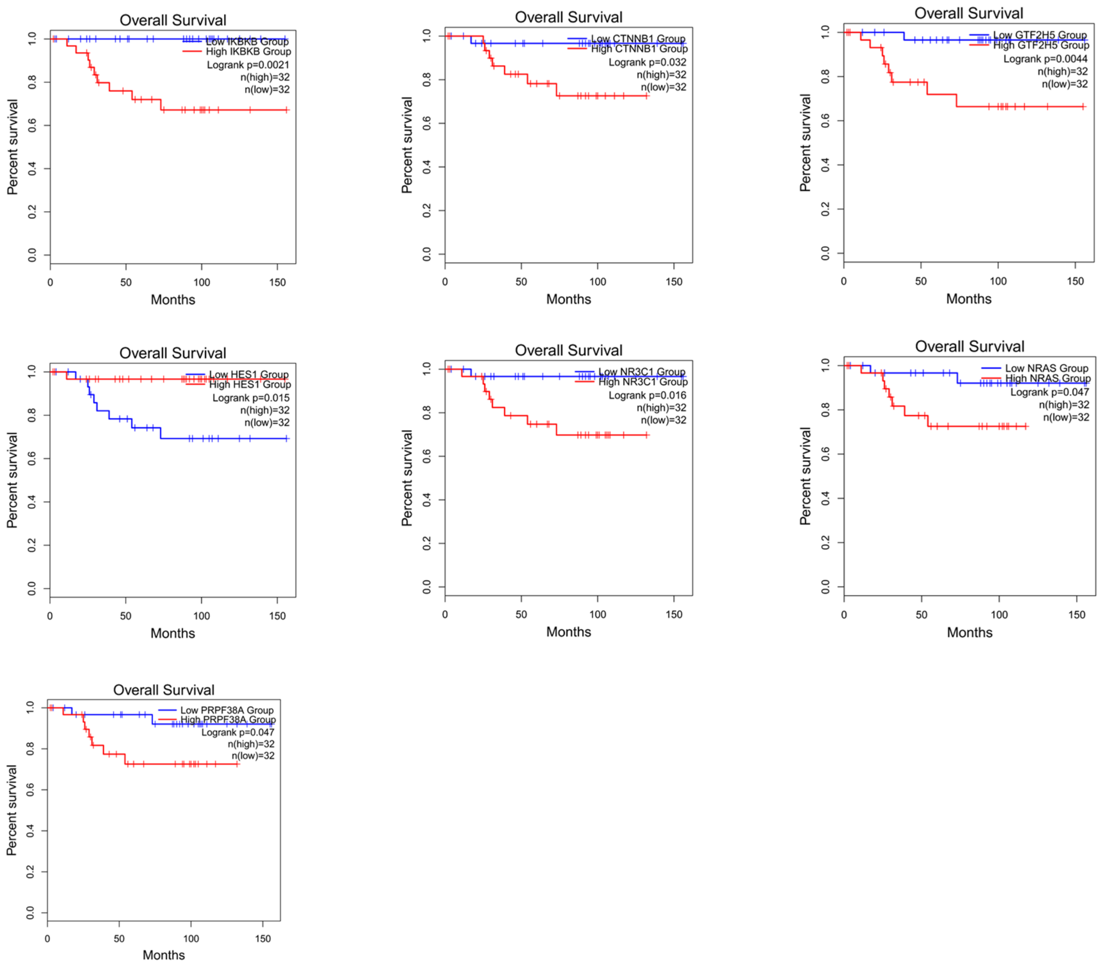

Figure S1: Survival analysis graph of 7 biomarker genes on KICH.

Literature validation of new biomarker genes on two datasets was performed. The results are shown in Table S1.

Table S1: The table shows the new biomarker genes identified in the two datasets and the corresponding descriptions.

	ACC	
Novel significant biomarker	Description	References
CDC42	Signaling pathways involved in mediating ACC [26].	Gara et al.(2015)

KAT5	Involved in the development of multiple cancers and tyrosine phosphorylation of KAT5 is defined as a key event in genomic and chromatin perturbation perception [27].	Kaidi et al.(2013)
APEX1	A small proportion of ACC is associated with aldosteronism, and APEX1 is involved in gene transcription of aldosterone synthase [28].	Mouat et al.(2019)
CDH2	CDH2 expression has prognostic significance in glioma [6].	Chen et al.(2018)
KAT2A	The first transcription-related KAT identified, significantly upregulated in many cancers, promotes tumor cell growth/cell proliferation [29].	Lin et al.(2022)
PARP1	Potential drug targets involved in the next generation of ACC [30].	Altieri et al.(2020)
TJP1	Involved in the regulation of LGR5, which reduces aldosterone production and affects the development of ACC [31].	Shaikh et al.(2015)
VAV2	New prognostic markers and drug targets for ACC [32]. Blocking VAV2 may be a new therapeutic approach to inhibit metastatic progression in patients with ACC [33].	Ruggiero et al. (2017)<br>Ruggiero et al(2017)
LRP6	The Wnt pathway co-receptor LRP6 is mainly expressed in benign adrenocortical tumors [34]. Inhibition of LRP6 expression contributes to the inhibition of ACC cell proliferation and induction of apoptosis [35].	Parviainen et al. (2013)<br>Zhu et al.(2017)
	KICH	
CTNNB1	Its mutational activation may be involved in the development of PICAN, which contains KICH, KIRC, and KIRP cancers [21].	Bruder et al.(2007)
GTF2H5	Low levels of GTF2H5 are associated with enhanced prognosis in high-grade serous ovarian cancer patients and may contribute to cisplatin sensitization [36].	Gayarre et al.(2016)
HES1	Known Notch signaling targets, increased and decreased levels of Notch signaling are associated with kidney cancer [37].	Mukherjee et al.(2019)

IKBKB	A regulator and target gene of NF-kB signaling, whose expression is significantly associated with poor prognosis and survival outcomes in kidney cancer [38].	Peri et al.(2013)
NR3C1	NR3C1 was identified as an epigenetically dysregulated gene in colorectal tumorigenesis [39]. midkine promotes breast cancer cell proliferation and migration through upregulation of NR3C1 expression and activation of NF-kB signaling pathway [40].	Lind et al. (2016)<br>Zhang et al.(2022)
NRAS	Mutational status of NRAS is an independent prognostic factor in metastatic melanoma [41], and has prognostic value in metastatic colorectal cancer [42].	Jakob et al. (2012)<br>Therkildsen et al.(2014)
PRPF38A	Potential biomarker candidates for osteosarcoma [43].	Chang et al.(2020)

Section S3

In this section, we will expand on the KEGG pathway enrichment analysis and GO function enrichment analysis on both datasets.

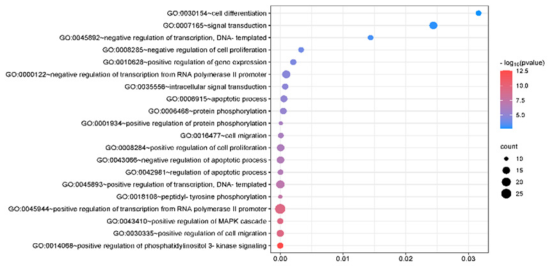

(a) ACC-GO-BP

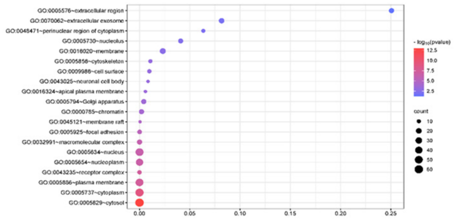

(b) ACC-GO-CC

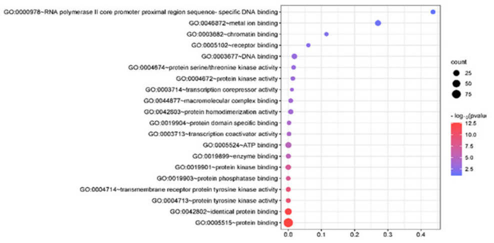

(c) ACC-GO-MF

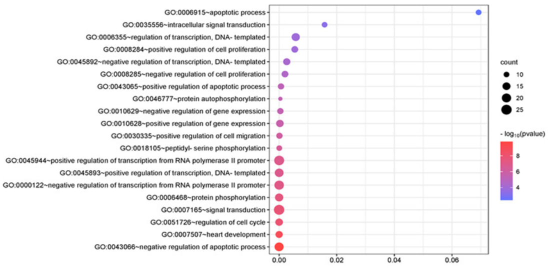

(d) KICH-GO-BP

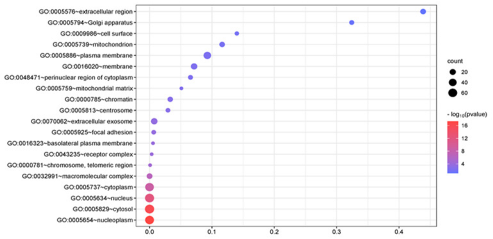

(e) KICH-GO-CC

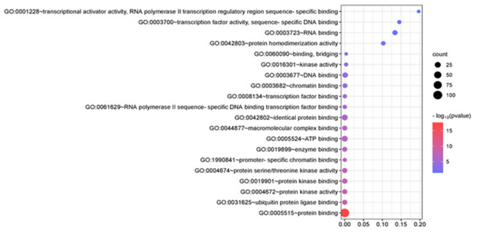

(f) KICH-GO-MF

Figure S2: KEGG pathway and GO function enrichment analysis. (a) ACC-GO-BP. (b) ACC-GO-CC. (c) ACC-GO-MF. (d) KICH-GO-BP. (e) KICH-GO-CC. (f) KICH-GO-MF.

The most important pathway in ACC is "Pathways in cancer", which indicates that our predicted driver genes are significantly associated with cancer. The most important pathway in ACC is "Pathways in cancer", which indicates that our predicted driver genes are significantly associated with cancer. We also predicted pathways associated with other cancers, such as "Proteoglycans in cancer" and "Ras signaling pathway" associated with lung cancer [44]. "PI3K-Akt signaling pathway" is associated with bladder metastatic cell carcinoma [45].

Positive regulation of RNA polymerase II promoter transcription, which is mainly enriched on GO-BP, can interact with Ago1 to positively affect gene expression in

cancer cells [46]. On GO-CC, it is mainly enriched in cytoplasm, cytosol and cytoplasmic membrane, etc. Some studies showed that the specific hydrolase activity in ACC was positively correlated with the activity of cytoplasm [47]. The large enrichment in protein binding to GO-MF indicates that the development of ACC is closely related to protein binding, especially the corresponding inhibitors of "protein tyrosine kinase activity" and "transmembrane receptor protein tyrosine kinase activity" are used as therapeutic agents for ACC [48].

The most important pathway in the KICH pathway is also the "Pathways in cancer". We also predicted pathways related to other cancers, such as "Human papillomavirus infection", which is a human papillomavirus (HPV) that causes cervical cancer as well as oral cancer, and the oncogenicity of these HPV types is mainly caused by the activity of oncoproteins E6 and E7, which impair the growth regulatory pathways and subsequently lead to cancer after slow mutation in the host genes accumulation leads to cancer [49]." MAPK signaling pathway", MAPK signaling pathway is associated with the development of many human diseases including Alzheimer's disease (AD), Parkinson's disease (PD), amyotrophic lateral sclerosis (ALS) and various types of cancer [50]." microRNAs in cancer", mRNAs are implicated in the regulation of almost all signaling circuits in cells and their dysregulation has been shown to play a crucial role in the development and progression of cancer, and these effects lead to the progressive transformation of normal cells into cancer cells [51].

Where GO-BP is predominantly enriched in signaling, the process of converting cellular proto-oncogenes into oncogenes can lead to over-activation of these signaling pathways, while inactivation of tumor suppressors eliminates key negative regulators of signaling, while interacting with PI3K-Akt and Ras-ERK pathways to dysregulated cancer signaling and generate many tumor cells [52]. On GO-CC, it is mainly enriched in the cell membrane, nucleus, nucleoplasm, and cytoplasm, and to a lesser extent in the chromosomes. Related studies suggest that the deletion of DNA on chromosomes may be a unique feature of KICH carcinoma [53]. Highly enriched in protein binding to GO-MF. Immunohistochemical analysis of Ksp-calmucin provides a reliable method for differentiating renal tumors from KICH carcinomas [54].

Section S4

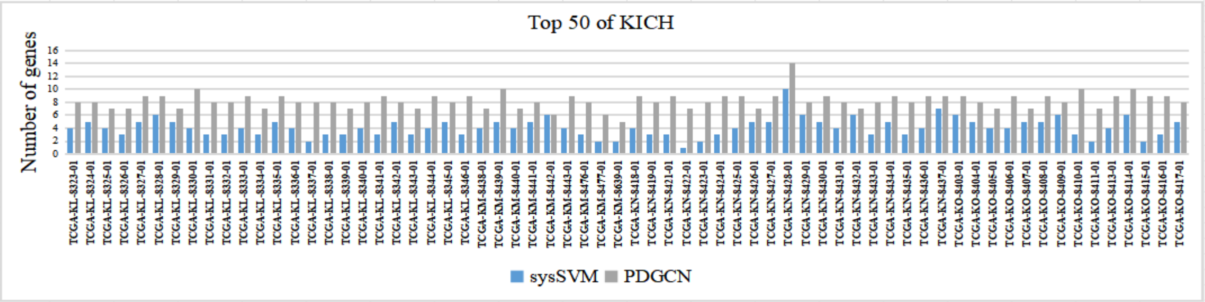

(a)

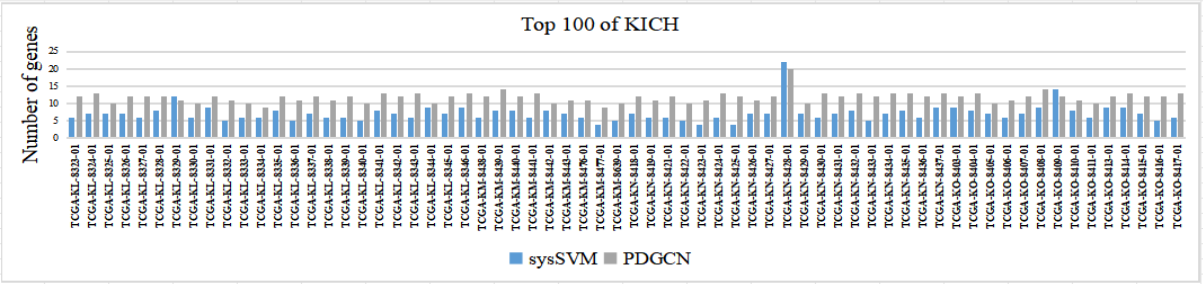

(b)

Figure S3: The comparison of PDGCN and sysSVM at individual level. The horizontal axis represents each sample and the vertical axis represents the number of genes in CGC: (a) Top 50 of KICH, and (b) Top 100 of KICH.

List of abbreviations in article

PDGCN (Personalized Drivers of GCN)

ACC (Adrenocortical Cancer)

KICH (Kidney Chromophobe)

TCGA (The Cancer Genome Atlas)

SNVs (Single Nucleotide Variations)

CNVs (Copy Number Variations)

CDGs (Cancer Driver genes)

DEGs (Differentially Expressed genes)

GCN (Graph Convolutional Network)

CRF (Conditional Random Field)

NCG (Network of Cancer Gene)

CGC (Cancer Gene Census)

COSMIC (the Catalogue Of Somatic Mutations In Cancer)

PPI (Protein-Protein Interaction Networks)

MC3 (Multiple Cancers)

ReLU (Rectified Linear Units)

ACC (Accuracy)

AUC (Area Under Curve)

AUPR (Area under Precision-Recall curve)

P (Precision)

R (Recall)

GEPIA2 (Gene Expression Profiling Interactive Analysis)

DAVID (the Database for Annotation,Visualization and Integrated Discovery)

KEGG (Kyoto Encyclopedia of Genes and Genomes)

GO (Gene Ontology)

GO-BP (Gene Ontology-Biological Processes)

GO-CC (Gene Ontology-Cellular Components)

GO-MF (Gene Ontology-Molecular Functions)

References

[1] Liang H, Lin Z, Ye Y, et al. ARRB2 promotes colorectal cancer growth through triggering WTAP[J]. Acta biochimica et biophysica Sinica, 2021, 53(1): 85-93.

[2] Jiang T, Yu J T, Wang Y L, et al. The genetic variation of ARRB2 is associated with late-onset Alzheimer's disease in Han Chinese[J]. Current Alzheimer Research, 2014, 11(4): 408-412.

[3] Zhou B, Song H, Xu W, et al. The Comprehensive Analysis of Hub Gene ARRB2 in Prostate Cancer[J]. Disease Markers, 2022, 2022.

[4] Ma T, Zhao Y, Wei K, et al. MicroRNA-124 functions as a tumor suppressor by regulating CDH2 and epithelial-mesenchymal transition in non-small cell lung cancer[J]. Cellular Physiology and Biochemistry, 2016, 38(4): 1563-1574.

[5] Miao J, Wang W, Wu S, et al. miR-194 suppresses proliferation and migration and promotes apoptosis of osteosarcoma cells by targeting CDH2[J]. Cellular Physiology and Biochemistry, 2018, 45(5): 1966-1974.

[6] Chen Q, Cai J, Jiang C. CDH2 expression is of prognostic significance in glioma and predicts the efficacy of temozolomide therapy in patients with glioblastoma[J]. Oncology letters, 2018, 15(5): 7415-7422.

[7] Cao L, Cheng H, Jiang Q, et al. APEX1 is a novel diagnostic and prognostic biomarker for hepatocellular carcinoma[J]. Aging (Albany NY), 2020, 12(5): 4573.

[8] Tummanatsakun D, Proungvitaya T, Roytrakul S, et al. Serum apurinic/apyrimidinic endodeoxyribonuclease 1 (APEX1) level as a potential biomarker of cholangiocarcinoma[J]. Biomolecules, 2019, 9(9): 413.

[9] Yang J, Yang D, Cogdell D, et al. APEX1 gene amplification and its protein overexpression in osteosarcoma: correlation with recurrence, metastasis, and survival[J]. Technology in cancer research & treatment, 2010, 9(2): 161-169.

[10] Li J, Feng Q, Qi Y, et al. PPARGC1A is upregulated and facilitates lung cancer metastasis[J]. Experimental cell research, 2017, 359(2): 356-360.

[11] Xiao X, Wang W, Li Y, et al. HSP90AA1-mediated autophagy promotes drug resistance in osteosarcoma[J]. Journal of Experimental & Clinical Cancer Research, 2018, 37(1): 1-13.

[12] Okino K, Nagai H, Hatta M, et al. Up-regulation and overproduction of DVL-1, the human counterpart of the Drosophila dishevelled gene, in cervical squamous cell carcinoma[J]. Oncology reports, 2003, 10(5): 1219-1223.

[13] Nagahata T, Shimada T, Harada A, et al. Amplification, up-regulation and over-expression of DVL-1, the human counterpart of the Drosophila disheveled gene, in primary breast cancers[J]. Cancer science, 2003, 94(6): 515-518.

[14] Smith M J, O'Sullivan J, Bhaskar S S, et al. Loss-of-function mutations in SMARCE1 cause an inherited disorder of multiple spinal meningiomas[J]. Nature genetics, 2013, 45(3): 295-298.

[15] Wang P, Xie M, Yang D, et al. Integrative multi-omics analysis reveals the landscape of Cyclin-Dependent Kinase (CDK) family genes in pan-cancer[J]. 2022.

[16] Caliskan A, Andac A C, Arga K Y. Novel molecular signatures and potential

therapeutics in renal cell carcinomas: Insights from a comparative analysis of subtypes[J]. Genomics, 2020, 112(5): 3166-3178.
[17] Zhou L, Yin B, Liu Y, et al. Mechanism and function of decreased FOXO1 in renal cell carcinoma[J]. Journal of surgical oncology, 2012, 105(8): 841-847.
[18] Kojima T, Shimazui T, Horie R, et al. FOXO1 and TCF7L2 genes involved in metastasis and poor prognosis in clear cell renal cell carcinoma[J]. Genes, Chromosomes and Cancer, 2010, 49(4): 379-389.
[19] Xu J, Perecman A, Wiggins A, et al. MetastamiRs in Renal Cell Carcinoma: An Overview of MicroRNA Implicated in Metastatic Kidney Cancer[J]. Exon Publications, 2022: 71-93.
[20] Erdem M, Erdem S, Sanli O, et al. Up-regulation of TGM2 with ITGB1 and SDC4 is important in the development and metastasis of renal cell carcinoma[C]//Urologic Oncology: Seminars and Original Investigations. Elsevier, 2014, 32(1): 25. e13-25. e20.
[21] Bruder E, Moch H, Ehrlich D, et al. Wnt signaling pathway analysis in renal cell carcinoma in young patients[J]. Modern pathology, 2007, 20(12): 1217-1229.2
[22] Cui J, Yuan Y, Shanmugam M K, et al. MicroRNA-196a promotes renal cancer cell migration and invasion by targeting BRAM1 to regulate SMAD and MAPK signaling pathways[J]. International journal of biological sciences, 2021, 17(15): 4254.
[23] Dirim A, Haberal A N, Goren M R, et al. VEGF, COX-2, and PCNA expression in renal cell carcinoma subtypes and their prognostic value[J]. International urology and nephrology, 2008, 40(4): 861-868.
[24] Altintas E, Kaynar M, Celik Z E, et al. Expression of Ring Box-1 protein and its relationship with Fuhrman grade and other clinical-pathological parameters in renal cell cancer[C]//Urologic Oncology: Seminars and Original Investigations. Elsevier, 2020, 38(1): 6. e17-6. e22.
[25] Chen C, Chi H, Min L, Junhua Z. Downregulation of guanine nucleotide-binding protein beta 1 (GNB1) is associated with worsened prognosis of clearcell renal cell carcinoma and is related to VEGF signaling pathway. J BUON. 2017;22:1441–6.
[26] Gara S K, Wang Y, Patel D, et al. Integrated genome-wide analysis of genomic changes and gene regulation in human adrenocortical tissue samples[J]. Nucleic acids research, 2015, 43(19): 9327-9339.
[27] Kaidi A, Jackson S P. KAT5 tyrosine phosphorylation couples chromatin sensing to ATM signalling[J]. Nature, 2013, 498(7452): 70-74.
[28] Mouat I C, Omata K, McDaniel A S, et al. Somatic mutations in adrenocortical carcinoma with primary aldosteronism or hyperreninemic hyperaldosteronism[J]. Endocrine-related cancer, 2019, 26(2): 217-225.
[29] Lin S, Qiu L, Liang K, et al. KAT2A/E2F1 Promotes Cell Proliferation and Migration via Upregulating the Expression of UBE2C in Pan-Cancer[J]. Genes, 2022, 13(10): 1817.
[30] Altieri B, Ronchi C L, Kroiss M, et al. Next-generation therapies for adrenocortical carcinoma[J]. Best Practice & Research Clinical Endocrinology & Metabolism, 2020, 34(3): 101434.
[31] Shaikh L H, Zhou J, Teo A E D, et al. LGR5 activates noncanonical Wnt signaling and inhibits aldosterone production in the human adrenal[J]. The Journal of Clinical

Endocrinology & Metabolism, 2015, 100(6): E836-E844.
[32] Ruggiero C, Lalli E. VAV2: a novel prognostic marker and a druggable target for adrenocortical carcinoma[J]. Oncotarget, 2017, 8(51): 88257.
[33] Ruggiero C, Doghman-Bouguerra M, Sbiera S, et al. Dosage-dependent regulation of VAV2 expression by steroidogenic factor-1 drives adrenocortical carcinoma cell invasion[J]. Science Signaling, 2017, 10(469): eaal2464.
[34] Parviainen H, Schrade A, Kiiveri S, et al. Expression of Wnt and TGF-β pathway components and key adrenal transcription factors in adrenocortical tumors: association to carcinoma aggressiveness[J]. Pathology-Research and Practice, 2013, 209(8): 503-509.
[35] Zhu Y, Wang M, Zhao X, et al. Rottlerin as a novel chemotherapy agent for adrenocortical carcinoma[J]. Oncotarget, 2017, 8(14): 22825.
[36] Gayarre J, Kamieniak M M, Cazorla-Jiménez A, et al. The NER-related gene GTF2H5 predicts survival in high-grade serous ovarian cancer patients[J]. Journal of Gynecologic Oncology, 2016, 27(1).
[37] Mukherjee M, Fogarty E, Janga M, et al. Notch signaling in kidney development, maintenance, and disease[J]. Biomolecules, 2019, 9(11): 692.
[38] Peri S, Devarajan K, Yang D H, et al. Meta-analysis identifies NF-κB as a therapeutic target in renal cancer[J]. PloS one, 2013, 8(10): e76746.
[39] Lind G E, Kleivi K, Meling G I, et al. ADAMTS1, CRABP1, and NR3C1 identified as epigenetically deregulated genes in colorectal tumorigenesis[J]. Analytical Cellular Pathology, 2006, 28(5-6): 259-272.
[40] Zhang L, Song L, Xu Y, et al. Midkine promotes breast cancer cell proliferation and migration by upregulating NR3C1 expression and activating the NF-κB pathway[J]. Molecular Biology Reports, 2022, 49(4): 2953-2961.
[41] Jakob J A, Bassett Jr R L, Ng C S, et al. NRAS mutation status is an independent prognostic factor in metastatic melanoma[J]. Cancer, 2012, 118(16): 4014-4023.
[42] Therkildsen C, Bergmann T K, Henrichsen-Schnack T, et al. The predictive value of KRAS, NRAS, BRAF, PIK3CA and PTEN for anti-EGFR treatment in metastatic colorectal cancer: A systematic review and meta-analysis[J]. Acta oncologica, 2014, 53(7): 852-864.
[43] Chang S, Cao Y. Differentially expressed genes SNRPC and PRPF38A are potential biomarkers candidates for osteosarcoma[J]. 2020.
[44] Liu Y, Ni R, Zhang H, et al. Identification of feature genes for smoking-related lung adenocarcinoma based on gene expression profile data[J]. OncoTargets and therapy, 2016, 9: 7397.
[45] Sathe A, Nawroth R. Targeting the PI3K/AKT/mTOR Pathway in Bladder Cancer Methods Mol Biol 2018;
1665:335–350.
[46] Huang V, Zheng J, Qi Z, et al. Ago1 Interacts with RNA polymerase II and binds to the promoters of actively transcribed genes in human cancer cells[J]. PLoS genetics, 2013, 9(9): e1003821.
[47] Papadopoulos D, Gröndal S, Rydström J, et al. Levels of cytochrome P-450, steroidogenesis and microsomal and cytosolic epoxide hydrolases in normal human

adrenal tissue and corresponding tumors[J]. Cancer biochemistry biophysics, 1992, 12(4): 283-291.
[48] Patalano A, Brancato V, Mantero F. Adrenocortical cancer treatment[J]. Hormone Research in Paediatrics, 2009, 71(Suppl. 1): 99-104.
[49] Schiffman M, Doorbar J, Wentzensen N, et al. Carcinogenic human papillomavirus infection[J]. Nature reviews Disease primers, 2016, 2(1): 1-20.
[50] Kim E K, Choi E J. Pathological roles of MAPK signaling pathways in human diseases[J]. Biochimica et Biophysica Acta (BBA)-Molecular Basis of Disease, 2010, 1802(4): 396-405.
[51] Di Leva G, Garofalo M, Croce C M. MicroRNAs in cancer[J]. Annual review of pathology, 2014, 9: 287.
[52] Gomperts B D, Tatham P E R, Kramer I M. Signal transduction[M]. Gulf Professional Publishing, 2002.
[53] Akhtar M, Kardar H, Linjawi T, et al. Chromophobe cell carcinoma of the kidney[J]. The American journal of surgical pathology, 1995, 19(11): 1245-1256.
[54] Mazal P R, Exner M, Haitel A, et al. Expression of kidney-specific cadherin distinguishes chromophobe renal cell carcinoma from renal oncocytoma[J]. Human pathology, 2005, 36(1): 22-28.
